# IFNγ^+^ NKT-like cells are associated with increased incidence of atrial fibrillation in elderly women

**DOI:** 10.1093/ehjopen/oeaf063

**Published:** 2025-06-05

**Authors:** Kari Anne Sveen, J Gustav Smith, Daniel Engelbertsen, Alexandru Schiopu, Andreas Edsfeldt, Gunnar Engström, Isabel Goncalves, Jan Nilsson, Harry Björkbacka, Eva Bengtsson

**Affiliations:** Department of Endocrinology, Morbid Obesity and Preventive Medicine, Oslo University Hospital, Trondheimsveien 235, 0424 Oslo, Norway; Department of Cardiology, Clinical Sciences, Lund University and Skåne University Hospital, 221 85 Lund, Sweden; The Wallenberg Laboratory/Department of Molecular and Clinical Medicine, Institute of Medicine, Gothenburg University and The Department of Cardiology, Sahlgrenska University Hospital, 413 45 Gothenburg, Sweden; Wallenberg Center for Molecular Medicine, Lund University, Klinikgatan 32, 221 84 Lund, Sweden; Lund University Diabetes Center, Lund University, Jan Waldenströms gata 35, 214 28 Malmö, Sweden; Lund University Diabetes Center, Lund University, Jan Waldenströms gata 35, 214 28 Malmö, Sweden; Department of Clinical Sciences Malmö, Lund University, Jan Waldenströms gata 35, 214 28 Malmö, Sweden; Lund University Diabetes Center, Lund University, Jan Waldenströms gata 35, 214 28 Malmö, Sweden; Department of Translational Medicine, Lund University, Jan Waldenströms gata 35, 214 28 Malmö, Sweden; Department of Internal Medicine, Skåne University Hospital, Entrégatan 7, 221 85 Lund, Sweden; N. Simionescu, Institute of Cellular Biology and Pathology, B. P. Hasdeu Str. 8, 050568 Bucharest, Romania; Wallenberg Center for Molecular Medicine, Lund University, Klinikgatan 32, 221 84 Lund, Sweden; Lund University Diabetes Center, Lund University, Jan Waldenströms gata 35, 214 28 Malmö, Sweden; Department of Clinical Sciences Malmö, Lund University, Jan Waldenströms gata 35, 214 28 Malmö, Sweden; Department of Cardiology, Skåne University Hospital, 205 02 Malmö, Sweden; Department of Clinical Sciences Malmö, Lund University, Jan Waldenströms gata 35, 214 28 Malmö, Sweden; Lund University Diabetes Center, Lund University, Jan Waldenströms gata 35, 214 28 Malmö, Sweden; Department of Clinical Sciences Malmö, Lund University, Jan Waldenströms gata 35, 214 28 Malmö, Sweden; Department of Cardiology, Skåne University Hospital, 205 02 Malmö, Sweden; Lund University Diabetes Center, Lund University, Jan Waldenströms gata 35, 214 28 Malmö, Sweden; Department of Clinical Sciences Malmö, Lund University, Jan Waldenströms gata 35, 214 28 Malmö, Sweden; Lund University Diabetes Center, Lund University, Jan Waldenströms gata 35, 214 28 Malmö, Sweden; Department of Clinical Sciences Malmö, Lund University, Jan Waldenströms gata 35, 214 28 Malmö, Sweden; Lund University Diabetes Center, Lund University, Jan Waldenströms gata 35, 214 28 Malmö, Sweden; Department of Clinical Sciences Malmö, Lund University, Jan Waldenströms gata 35, 214 28 Malmö, Sweden; Department of Biomedical Science, Faculty of Health and Society, Malmö University, Jan Waldenströms gata 25, 214 28 Malmö, Sweden; Biofilms—Research Center for Biointerfaces, Malmö University, Per Albin Hanssons väg 35, 214 32 Malmö, Sweden

**Keywords:** Incident atrial fibrillation, T cell, NKT-like cell

## Abstract

**Aims:**

T cells are present in atrial tissue from atrial fibrillation (AF) patients. However, prospective studies of T cells and AF development are few. The current aim was to investigate if T-cell subsets are associated with the risk of developing AF.

**Methods and results:**

T-cell subsets, measured by flow cytometry of cryopreserved mononuclear leucocytes isolated from blood at baseline, were analysed for associations of incident AF in 669 subjects from a population-based cohort. Subjects were followed for incidence of AF for 18.6 (11.5–21.7) years during which 145 subjects were diagnosed with AF. Incident AF cases had higher levels of CD3^+^CD56^+^ NKT-like cells. No differences in CD3^+^, CD3^+^CD4^+^, CD3^+^CD8, Th1, Th2, or regulatory T cells between incident AF cases and non-cases were observed. Women had higher levels of NKT-like cells than men. High numbers of NKT-like cells were associated with an increased risk of developing AF in women [HR (95% CI) 1.88 (1.10–3.23) above vs. below median], but not in men or in the total cohort. The majority of NKT-like cells were IFNγ^+^ after stimulation. High numbers of IFNγ^+^ NKT-like cells were associated with increased risk for developing AF in women. Median fluorescence intensity of IFNγ for NKT-like cells was higher in cases of incident AF in women, but not in the total cohort or in men.

**Conclusion:**

High levels of IFNγ^+^ NKT-like cells in blood are associated with increased risk of incident AF in women, supporting a role for T cells in development of AF and emphasizing sex differences in this context.

## Graphical Abstract

**Graphical Abstract:**
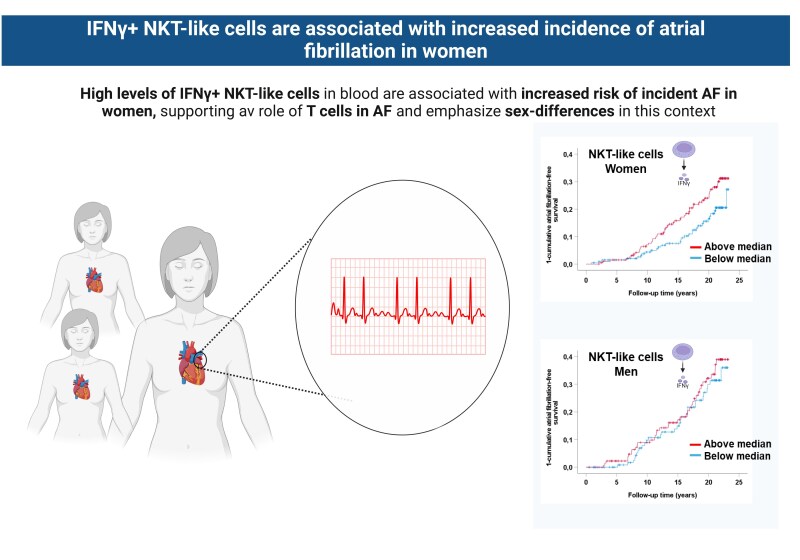

## Introduction

Atrial fibrillation (AF) is the most common chronic arrhythmia linked to a high morbidity and mortality.^1^ Atrial fibrillation’s pathogenic processes including structural and electrical remodelling of the atria are still not fully understood, although escalating data suggest a link between inflammation and AF.^2,3^ Different systemic inflammatory markers have been associated with AF,^2–4^ sparking the debate of an innate immune response as a cause of AF in humans or if it merely signals ongoing inflammation. Experimental studies have demonstrated causal roles for monocyte migration to the atria^5^ and inflammasome activation in cardiomyocytes^6^ in AF in mice. In line with this, monocyte counts in peripheral blood are associated with increased risk of developing AF in humans.^7^ Importantly, atrial tissue from patients contains more macrophages^8^ and mononuclear phagocyting cells,^5^ and gene expression analysis of these cells revealed an up-regulation of inflammatory and IFNγ-signalling pathways.^5^ In addition to macrophages, atrial tissue from AF patients has higher levels of CD3^+^ T cells.^8,9^ Recent data comparing immune cells from atria vs. epicardial adipose tissue from patients revealed an expression of genes associated with T-cell activation in tissue-resident memory T cells in atria, of which *IFNG* was one of the most up-regulated genes.^10^ These cells or IFNγ alone modulated ion channel expression and calcium handling in cardiomyocytes *in vitro*, indicating changes in electrical properties of the cardiomyocytes.^10^ Together, these data suggest that signalling between T cells and macrophages or between T cells and cardiomyocytes, where IFNγ is one of the mediators, promotes AF. However, prospective studies of T-cell subsets in blood and their association with development of AF are few.^11^

T cells are part of the adaptive immune responses comprising cytotoxic CD3^+^CD8^+^ T cells, as well as CD3^+^CD4^+^ T cells, the latter separated into IFNγ^+^ Th1, IL-4^+^ Th2, and regulatory T cells. A distinct population is CD3^+^CD56^+^ NKT-like cells, which are T cells expressing markers of natural killer cells including CD56,^12^ thus sharing receptors with both adaptive and innate immune cells. By analogy, NKT-like cells are divided into CD8^+^ and CD4^+^ NKT-like cells. NKT-like cells have cytotoxic properties and are important in cytokine production. They secrete the inflammatory cytokines TNFα and IFNγ, having the ability to produce more IFNγ than ordinary CD8^+^ T cells.^12^

In the present study, we investigated T-cell subsets from cryopreserved mononuclear leucocytes isolated from blood at baseline and their associations with development of AF in 669 subjects from a population-based cohort. We found that IFNγ^+^ CD3^+^CD56^+^ NKT-like cells are associated with increased risk of developing AF in women, independently of common risk factors for AF. Furthermore, the median fluorescence intensity of IFNγ in NKT-like cells was higher in women with incident AF.

## Methods

### Study population

The Malmö Diet and Cancer cohort (MDC) is a population-based prospective cohort including 28 449 participants enrolled 1991–96. Between October 1991 and February 1994, every other participant was invited to take part in a substudy of the epidemiology of cardiovascular disease (MDC-CC). Participants were followed from baseline examination until a first event of AF, emigration from Sweden, or death until 31 December 2014. Baseline samples of mononuclear leucocytes were isolated from blood and stored in medium with autologous serum and DMSO at −140°C as previously described.^13^ In the present analysis, mononuclear leucocyte samples were analysed by flow cytometry in a random subsample of 700 individuals from MDC-CC aged 63–68 years.^13^ After exclusion of individuals with prevalent AF or missing information for lymphocytes, the study included 524 non-cases and 145 incident AF cases. The median follow-up was 18.6 (11.5–21.7) years. Information of baseline characteristics were collected from clinical examination or self-administered questionnaires.^14^ The study was approved by the Ethics Committee at Lund University (LU 51-90 and LU 532-2006). All participants gave informed consent. The study complies with the Declaration of Helsinki.

### Clinical events

AF [International Classification of Diseases (ICD) codes: 427.92 for ICD-8, 427D for ICD-9, and I48 for ICD-10] was identified linking Swedish personal identification numbers with national registers (Swedish Hospital Discharge Register and the Cause of Death Registry in Sweden).^15^ Diagnoses were validated by examining electrocardiograms and patient records in randomly selected samples from the MDC cohort. Electrocardiograms were available in 98% of validation samples, and AF was confirmed in 95 out of 98 available electrocardiograms.^15^ Coronary events included subjects with ICD codes 410, 414 (ICD-9), I21, and I25 (ICD-10) and strokes included subjects with ICD codes 430, 431, 434, and 436 (ICD-9), and I60-61 and I63-64 (ICD-10).

### Flow cytometry

T-cell subsets were analysed by flow cytometry of cryopreserved mononuclear leucocytes isolated from blood at baseline as described previously.^13,16^ Briefly, cells were carefully thawed and stimulated with phorbol 12-myristate 13-acetate (PMA), ionomycin and brefeldin to enable measurement of IFNγ and IL-4 expressing cells. Cells were stained with anti-CD56-biotin, anti-CD3-PE/Cy7, anti-CD4-PB, and anti-CD8-AF700, rinsed and incubated with streptavidin-PerCP. Cells were then incubated with Fix/Perm solution and permeabilization buffer, and subsequently incubated with anti-IFN-γ-PE and anti-IL-4-APC. Regulatory T cells (CD45^+^CD3^+^CD4^+^CD25^+^FoxP3^+^) were stained as described previously.^17^ All antibodies were from BioLegend. Absolute cell numbers in blood were calculated by multiplying percentages of gated lymphocyte populations with counts obtained from a blood cell count analysis, using a Sysmex K-1000 with data unit DA 1000 (TOA Medical Electronics Co). Samples were run on ADP-Cyan flow cytometer (Beckman Coulter, Sweden), and analyses were performed with FlowJo 7.5.5 (Treestar Inc., USA). Compensation beads were used to compensate fluorescent signals, and FMO control samples were used to set gate boundaries. An overview of the gating strategy is presented in Supplementary material online, *Figure S1*. Regulatory T cells (CD45^+^CD3^+^CD4^+^CD25^+^FoxP3^+^) were gated as described previously.^17^ Percentages T cells and NKT-like cells are expressed as % of lymphocytes. Percentages of Th1, Th2, and Tregs are expressed as % of CD4^+^ T cells.

The repertoire of innate like T cells comprise NKT-type I, which recognize lipid antigens (α-GalCer) presented by CD1d, NKT-type II recognizing antigens presented by CD1d but with a more varied TCR, and the CD3^+^CD56^+^ NKT-like cells with a diverse TCR.^12^ In the present paper, we analysed the latter population, CD3^+^CD56^+^ NKT-like cells, where only a very minor fraction (1.3%) was positive for CD1d/α-GalCer tetramers.^13^ Furthermore, the majority of CD1d/α-GalCer tetramer-stained cells were CD56^−^ and IFNγ^−^, confirming that the current analysis is performed on NKT-like cells.^13^

### IFNγ secretion of mononuclear cells

Mononuclear cells were stimulated with anti-CD3/CD28 beads for 72 h, and cytokine release in medium were analysed by Multiplex immunoassay (MesoScale Discovery) as described previously.^16^

### Statistics

The distributions of variables were determined by histograms and skewness and kurtosis measurements. Comparison of non-cases and incident AF cases were assessed by *t*-test or Mann–Whitney *U* test for continuous data and χ^2^ test for categorical data. Correlations were analysed using Spearman’s rho correlation coefficient. Prospective associations of NKT-like cells (standardized variable or below and above median) with incidence of AF were analysed by Kaplan–Meier curves and log-rank test. Hazard ratios and 95% confidence intervals of NKT-like cells (standardized variable or divided in above or below median) for incidence of AF were analysed by Cox proportional hazard regression analysis adjusting for age, sex, systolic blood pressure, current smoking, diabetes, body mass index, triglycerides, HDL, glomerular filtration rate, prevalent coronary event or stroke, and presence of carotid plaque at baseline. A sensitivity analysis excluding subjects with prevalent coronary events or strokes was performed using Cox regression analysis adjusting for confounders as above. In an additional Cox regression analysis, incident major adverse cardiac events prior incident AF were censored, where confounders were adjusted for as above. The confounders were selected based on significant differences in baseline characteristics between non-cases and incident AF cases and known risk factors and comorbidities of AF. Non-normally distributed variables were logarithmically transformed. Analyses were performed using SPSS (IBM SPSS version 28) and GraphPad Prism 9.

## Results

### The Malmö Diet and Cancer cohort

The present cohort included baseline samples from 700 individuals from the MDC Cardiovascular cohort. After excluding prevalent cases of AF and missing values, the present cohort included 669 subjects: 524 non-cases and 145 incident AF cases, the latter developing AF during the follow-up period. Incident AF cases had higher BMI than non-AF subjects. In addition, incident AF cases displayed trends towards higher systolic blood pressure, lower HDL, higher glucose, and included more men, but less smokers than non-AF cases (*Table 1*).

**Table 1:** Baseline characteristics of incident AF and non-AF subjects in the Malmö Diet and Cancer cardiovascular cohort

	Non-AF (*n* = 524)	Incident AF (*n* = 145)	*P*-value
Age, years	65.6 ± 1.1	65.6 ± 1.2	0.6
Sex, % men	39.3	46.2	0.1
Smoking, %	19.2	12.2	0.06
Diabetes mellitus,^a^ %	13.3	17.9	0.2
History of CE, %	2.3	1.4	0.5
History of stroke, %	0.4	1.4	0.2
History of heart failure, %	0.4	0	0.5
BMI, kg/m^2^	26.0 ± 3.8	27.2 ± 4.3	0.001
HbA1c, %	5.0 (4.6–5.3)	5.0 (4.7–5.4)	0.2
Glucose, mmol/L	5.0 (4.6–5.5)	5.1 (4.7–5.6)	0.06
Cholesterol, mmol/L	6.4 (5.7–7.1)	6.4 (5.7–7.2)	0.6
HDL, mmol/L	1.4 ± 0.4	1.3 ± 0.4	0.1
LDL, mmol/L	4.4 ± 1.0	4.3 ± 1.1	0.3
Triglycerides, mmol/L	1.3 (0.9–1.8)	1.4 (0.9–2.0)	0.2
Systolic blood pressure, mmHg	151 ± 20	153 ± 20	0.1
Diastolic blood pressure, mmHg	88 ± 9	89 ± 8	0.4
CRP, mg/L	1.6 (0.8–3.0)	1.6 (0.7–3.2)	1.0
eGFR, mL/min per 1.73 m^2^	66 (60–74)	67 (60–77)	0.3

Values are presented as mean ± SD, median and interquartile range (IQR), or percentage. *t*-Test or Mann–Whitney *U* for continuous data, χ^2^ or Fisher’s exact test for categorical data.

CE, major adverse coronary events; BMI, body mass index; eGFR, estimated glomerular filtration rate.

^a^History of diabetes mellitus, medication or fasting blood glucose > 6.1 mmol/L.

### NKT-like cells are higher in subjects with incident atrial fibrillation

**Figure 1:**
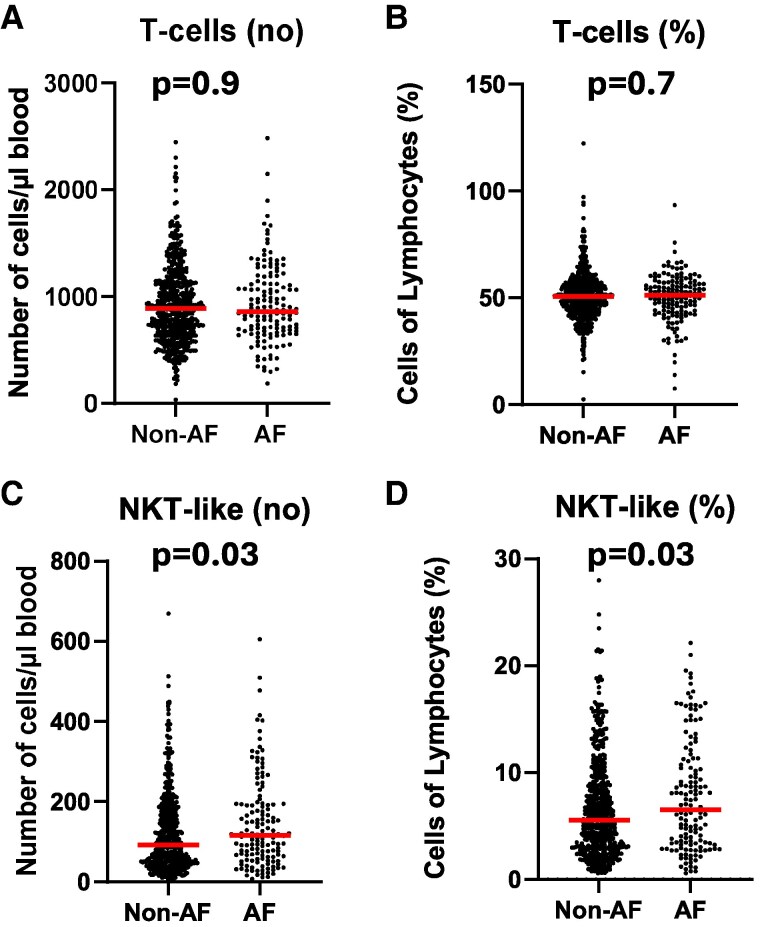
Higher levels of NKT-like cells in subjects who develop AF. CD3^+^CD56^−^ T cells (*A* and *B*) and CD3^+^CD56^+^ NKT-like cells (*C* and *D*) were analysed by flow cytometry of cryopreserved mononuclear leucocytes isolated from blood at baseline in individuals who developed atrial fibrillation (AF) during follow-up and in individuals who did not develop atrial fibrillation (non-AF).

T-cell subsets, analysed by flow cytometry of cryopreserved mononuclear leucocytes isolated from blood at baseline, were analysed for associations with incident AF. The number of CD3^+^ T cells or percentages of CD3^+^ T cells did not differ between incident AF cases and non-AF cases (*Figure 1A* and *B*). Furthermore, an analysis of CD4^+^ T-cell subsets including IFNγ^+^ Th1 cells, IL-4^+^ Th2 cells, and Foxp3^+^ regulatory T cells showed no significant differences between incident AF cases and non-cases (see Supplementary material online, *Figure S2*). In contrast, both numbers of CD3^+^CD56^+^ NKT-like cells and percentages of NKT-like cells were higher in cases with incident AF (*Figure 1C* and *D*) compared to non-AF subjects. In addition, numbers of CD8^+^ NKT-like cells displayed a trend towards higher levels in incident AF cases, whereas CD8^+^ or CD4^+^ T cells were not associated with incidence of AF (see Supplementary material online, *Table S1*).

### Associations of NKT-like cells with risk factors

Next, the association of NKT-like cells with risk factors for AF were analysed. Both numbers and percentages of NKT-like cells were inversely associated with age and HDL. Numbers of NKT-like cells were also associated with BMI, triglycerides and glomerular filtration rate (see Supplementary material online, *Table S2*). Interestingly, numbers of NKT-like cells were higher in women compared to men [106 (55–170) cells/µL blood vs. 88 (41–174) cells/µL blood, *P* = 0.04] (see Supplementary material online, *Table S3*).

### NKT-like cells are higher in women with incident atrial fibrillation

Men and women were then analysed separately. BMI was higher in women with incident AF compared to non-AF (see Supplementary material online, *Table S4*). Numbers of NKT-like cells were significantly higher in women with incident AF compared to non-cases [121 cells/µL blood (74–192) vs. 99 cells/µL blood (53–177); *P* = 0.039], but not in men [93 cells/µL blood (53–193) vs. 84 cells/µL blood (40–160), *P* = 0.24]. There were no significant sex differences in incidence of AF among the other T-cell subsets, although a trend towards higher levels of IFNγ^+^ Th1 cells was found in women with incident AF (see Supplementary material online, *Table S5*).

Analysing the association of NKT-like cells with risk factors separately in men and women (see Supplementary material online, *Tables S6* and *S7*) revealed an inverse association with HDL in both men and women. NKT-like cells were also associated with BMI in women, and with triglycerides in men.

### NKT-like cells are associated with increased risk of atrial fibrillation in women

Next, we analysed if there was an association of NKT-like cells (analysed as standardized continuous variable) and incidence of AF. Cox regression analysis showed that a high number of NKT-like cells was not significantly associated, but displayed a trend, towards increased risk of developing AF in the total cohort after adjusting for risk factors AF [HR (95% CI): 1.15 (0.98–1.36); standardized variable, *P* = 0.089]. In women, this association was significant, where high levels of NKT-like cells were associated with increased risk for AF [HR (95% CI): 1.26 (1.01–1.58); standardized variable, *P* = 0.040)], whereas no association was present in men (see Supplementary material online, *Table S8*). In addition, percentages of NKT-like cells were associated with increased risk of AF in the total cohort [HR (95% CI): 1.19 (1.01–1.41); standardized variable, *P* = 0.044]. Again, this association was stronger in women [HR (95% CI): 1.31 (1.02–1.70); standardized variable, *P* = 0.037], but was not present in men (see Supplementary material online, *Table S8*).

**Figure 2:**
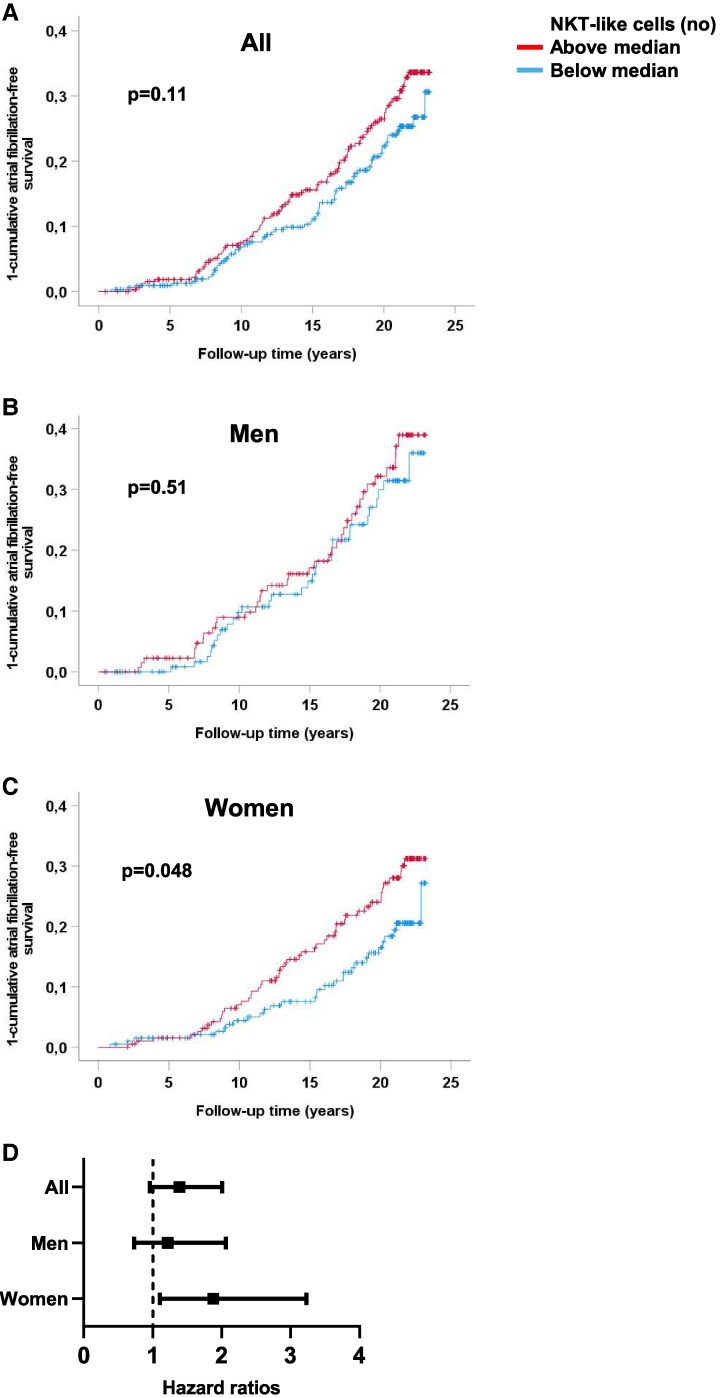
NKT-like cells are associated with incidence of atrial fibrillation in women. (*A–C*) Associations of numbers of NKT-like cells (above and below median) in blood and incident atrial fibrillation. (*D*) Hazard ratios (95% CI) of numbers of NKT-like cells above median vs. below median after adjusting for risk factors (age, sex, systolic blood pressure, current smoking, diabetes, body mass index, triglycerides, HDL, glomerular filtration rate, prevalent coronary event or stroke, and presence of carotid plaque).

NKT-like cells were then divided in high (above median) and low levels (below median) and analysed for risk of developing AF. A high number of NKT-like cells wer associated with incident AF in women, but not in men or in the total cohort (*Figure 2A–C*). Furthermore, the association of NKT-like cells with increased risk of AF in women was independent of risk factors (*Figure 2D*). To analyse if NKT-like cells in women were associated with increased risk of developing AF also in a population free of major cardiovascular events at baseline, we performed a sensitivity analysis excluding subjects with prevalent coronary events or strokes. The result showed that high levels (above median) of NKT-like cells were associated with increased risk of AF in women [HR (95% CI) 1.82 (1.06–3.12), *P* = 0.031] also in this population. Furthermore, to account for comorbidities over time that increase the risk for AF, subjects who suffered from coronary events after baseline, but prior AF, were censored in the Cox regression analysis. The analysis revealed that the association of high levels of NKT-like cells with risk of developing AF in women remained, and were even stronger, after censoring individuals with coronary events prior AF [HR (95% CI) above vs. below median: 2.07 (1.16–3.72), *P* = 0.014].

### IFNg^+^ NKT-like cells are associated with increased risk of atrial fibrillation in women

A large majority [88 (75–92%)] of activated NKT-like cells expressed IFNγ, and both CD4^+^ [84.1% (68.5–91.3)] and CD8^+^ [93.9% (86.7–96.6)] NKT-like cells were IFNγ-positive. In comparison, only 10.6% (6.0–17.4) of CD8^+^ T cells were IFNγ-positive. Furthermore, IFNγ median fluorescence intensity was higher in NKT-like cells than in CD8^+^ T cells or CD4^+^ T cells (see Supplementary material online, *Figure S3*). Stimulation of mononuclear cells with anti-CD3/CD28 revealed a strong correlation between numbers of NKT-like cells and amounts of secreted IFNγ (*r* = 0.24; *P* < 10^−9^). Both CD4^+^ and CD8^+^ NKT-like cells were similarly and strongly associated with IFNγ (*r* = 0.24; *P* < 10^−9^ and *r* = 0.23; *P* < 10^−8^, respectively). Not surprisingly, Th1 cells displayed the strongest correlation with IFNγ (*r* = 0.32; *P* < 10^−16^), whereas CD8^+^ T cells displayed a weaker association with secreted IFNγ (*r* = 0.15; *P* < 10^−4^).

We then analysed whether IFNγ^+^ NKT-like cells were associated with increased risk of developing AF. Similar to NKT-like cells; high levels of IFNγ^+^ NKT-like cells displayed a trend to increased risk for AF in the total cohort after adjusting for risk factors (*Table 2*). The association of IFNγ^+^ NKT-like cells with incident AF was significant in women, but not in men after adjusting for risk factors (*Table 2*). In addition, IFNγ median fluorescence intensity of NKT-like cells was higher in cases of incident AF in women, but not in men or in the total population (*Table 3*).

**Table 2:** Hazard ratios and 95% confidence intervals of incident AF above vs. below median of IFNγ^+^ NKT-like cells

		HR (95% CI)^a^	HR (95% CI)^a^	*P*
		Below median	Above median	
All	IFNγ^+^ NKT-like cells (no)	1	1.44 (1.00–2.09)	0.053
Men	IFNγ^+^ NKT-like cells (no)	1	1.16 (0.69–1.96)	0.57
Women	IFNγ^+^ NKT-like cells (no)	1	1.89 (1.10–3.24)	0.021

^a^Adjusted for age, sex, systolic blood pressure, current smoking, diabetes, BMI, triglycerides, HDL, eGFR, prevalent coronary event or stroke, and presence of carotid plaque.

**Table 3:** IFNγ median fluorescence intensity in NKT-like cells

		Non-AF	Incident AF	*P*-value
MFI IFNγ	All	380 (281–497)	383 (293–521)	0.31
MFI IFNγ	Men	401 (274–524)	357 (267–476)	0.26
MFI IFNγ	Women	377 (283–459)	418 (323–545)	0.020

## Discussion

In the present study we analysed T-cell subsets and the risk of developing AF in 669 randomly selected individuals from the general population. We found that high levels of CD3^+^CD56^+^NKT-like cells in blood were associated with increased risk of developing AF, whereas T cells, Th1, Th2, or regulatory T cells were not associated with incident AF. Women had higher levels of NKT-like cells, and an analysis of men and women separately showed that the association of NKT-like cells with incident AF was present in women, but not in men. The association of NKT-like cells with AF in women was independent of AF-associated risk factors. Furthermore, the association was also present in a population without cardiovascular comorbidities and remained when censoring subjects who had a coronary event before the onset of AF. Together, these results show that NKT-like cells are associated with increased risk for developing AF in women independently of known risk factors for AF.

NKT-like cells are high producers of IFNγ.^12^ In line, the vast majority of NKT-like cells in the present analysis were IFNγ-positive after activation. Similar results were presented by Bergstrom *et al*.,^18^ who found that 86% of CD8^+^CD56^+^ T cells were positive for IFNγ. In the present cohort, we found that IFNγ^+^ NKT-like cells were associated with an increased risk for developing AF in women. The HR ratios were similar, or slightly stronger, to HR of all NKT-like cells, which is consistent with that the majority of NKT-like cells were IFNγ^+^. Furthermore, median fluorescence intensity of NKT-like cells was higher in women with incident AF, strengthening the association with IFNγ expression in NKT-like cells with AF in women.

A recent study using single-cell RNA sequencing on immune cells and TCR sequencing suggested that tissue-resident memory T cells in the atria derive from tissue-resident memory T cells in the epicardial adipose tissue, as shown by shared expanded TCR clones.^10^ These T cells in the atria displayed an increased expression of T-cell activation genes, where *IFNG* was one of most up-regulated genes.^10^ Furthermore, these cells, or IFNγ alone, were able to alter electrical properties of induced cardiomyocytes *in vitro*. This study highlights a possible role of IFNγ-expressing T cells in AF and show that IFNγ has the ability to promote AF. The present data demonstrating that high numbers of IFNγ^+^ NKT-like cells in blood are associated with increased risk of developing AF support a role for IFNγ-expressing cells in AF, adding prospective data to this hypothesis.

Women and men display differences in incidence, risk factors, and outcomes regarding AF.^19^ Although these differences are known, the knowledge of how gender differences play a role in the development of AF is far from fully understood. Interestingly, the association of NKT-like cells in blood, and their IFNγ expression, with incident AF was present in women, but not in men or in the total cohort. Furthermore, IFNγ^+^ Th1 cells tended to be increased in incident AF cases in women, but not in men or in the total cohort. Although this sex difference is intriguing, it is known that there are differences in immune responses between sexes and this is also altered over the life course suggesting hormonal effects to be important.^20^ Oestradiol affects, directly or indirectly, many aspects of both adaptive and innate immunity, including altered production of IFNγ. However, whether oestradiol affects the number of NKT-like cells or their IFNγ levels has not been investigated. In our study, the women were post-menopausal. An ageing immune system is associated with an aberrant chronic low-grade pro-inflammatory state, which may occur to a greater extent in women partly due to the changed level of oestradiol at menopause.

The lifetime risk of AF is about one in 3–5 individuals after the age of 45 years.^21^ In the total MDC Cardiovascular cohort, the yearly incidence rate of AF was 0.74%.^14^ In the present substudy, consisting of 669 individuals from the same cohort, the yearly incidence rate was 1.34%. The higher incidence of AF in the present substudy is likely to be explained by the higher age of the individuals, and that subjects in the current substudy had more comorbidities including higher prevalence of diabetes and higher BMI than in the total study population.

The present study has some limitations, which are important to consider. Even though we show an association of NKT-like cells in blood with increased risk of developing AF, supporting a role for NKT-like cells in the development of AF, we cannot say if these cells, or their IFNγ secretion, indeed have a causal role. Furthermore, the association was performed on a cohort with a limited age span, which may limit the generalizability of the study. However, the age span is relevant considering the age of onset of AF.^22,23^ The strengths of the present study are the high number of individuals analysed by flow cytometry. Furthermore, the Swedish national registers using personal numbers minimize the potential of missing data of incident AF cases and gives valid data representing a broad spectrum of the population in Sweden. In addition, the case validity of the AF diagnosis of the cohort was previously shown to be very high.^15^ However, we cannot exclude that subjects with asymptomatic disease may have gone undetected.

In summary, we show that high levels of IFNγ^+^NKT-like cells in blood are associated with increased risk for developing AF in women. This study adds upon the importance of immune cells in AF and emphasizes gender differences in this context.

## Lead author biography


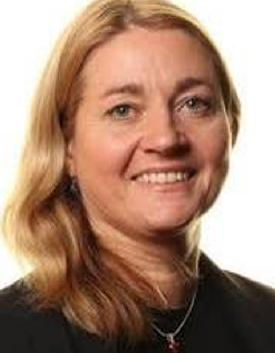


Kari Anne Sveen is a Clinical Scientist and a Medical doctor in Internal medicine and endocrinology. She has pursued a PhD at the University of Oslo in Norway, followed by a postdoctoral stay at CRC, Lund University in Sweden and University of Oslo under the Marie Skłodowska-Curie actions European Union Seventh Framework Programme for Research. Her research focuses on immunological mechanisms in cardiovascular- and metabolic diseases, with a special emphasis on the interplay of inflammation and atrial fibrillation.

## Supplementary Material

oeaf063_Supplementary_Data

## Data Availability

The data that support the findings of this study are available from the authors upon reasonable request, but, due to some of the data containing information that could compromise research participant privacy or consent, requests may require ethical review and full compliance to the general data protection regulation.
