# Supplementary material for: IFNγ+ NKT-like cells are associated with increased incidence of atrial fibrillation in elderly women
Source: Eur Heart J Open. 2025 Jun 5;5(3):oeaf063. doi: 10.1093/ehjopen/oeaf063 (PMC12198755; doi:10.1093/ehjopen/oeaf063)
Supplement: oeaf063_Supplementary_Data [file oeaf063_supplementary_data.pdf]

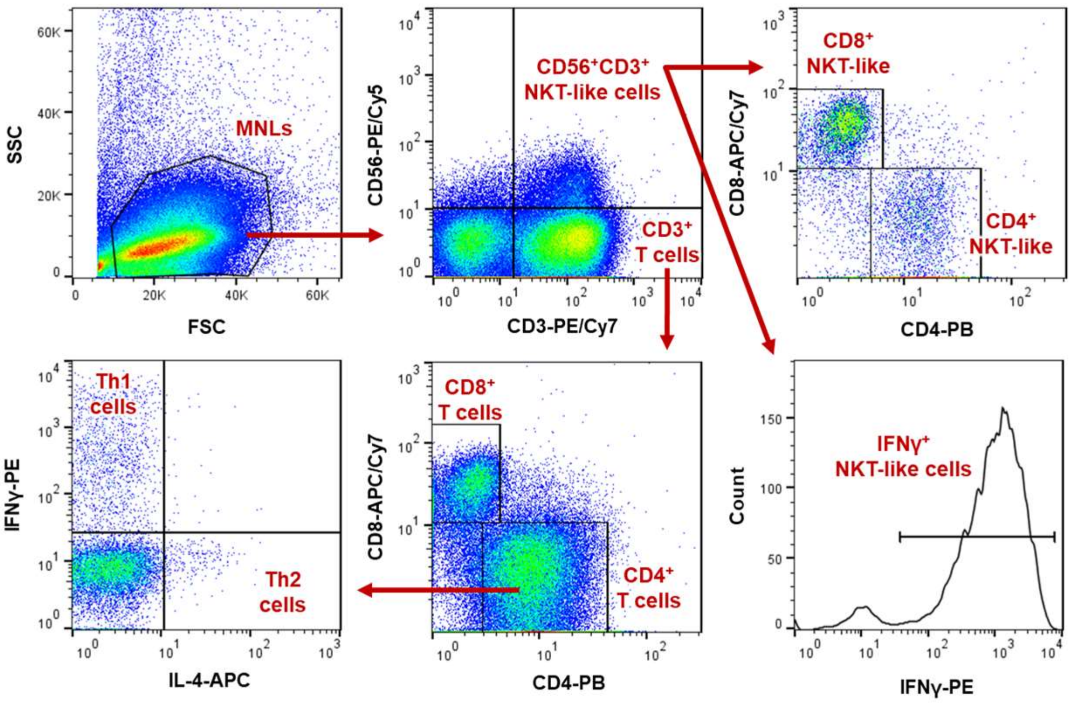

Figure S1. The gating strategy of T-cell subsets.

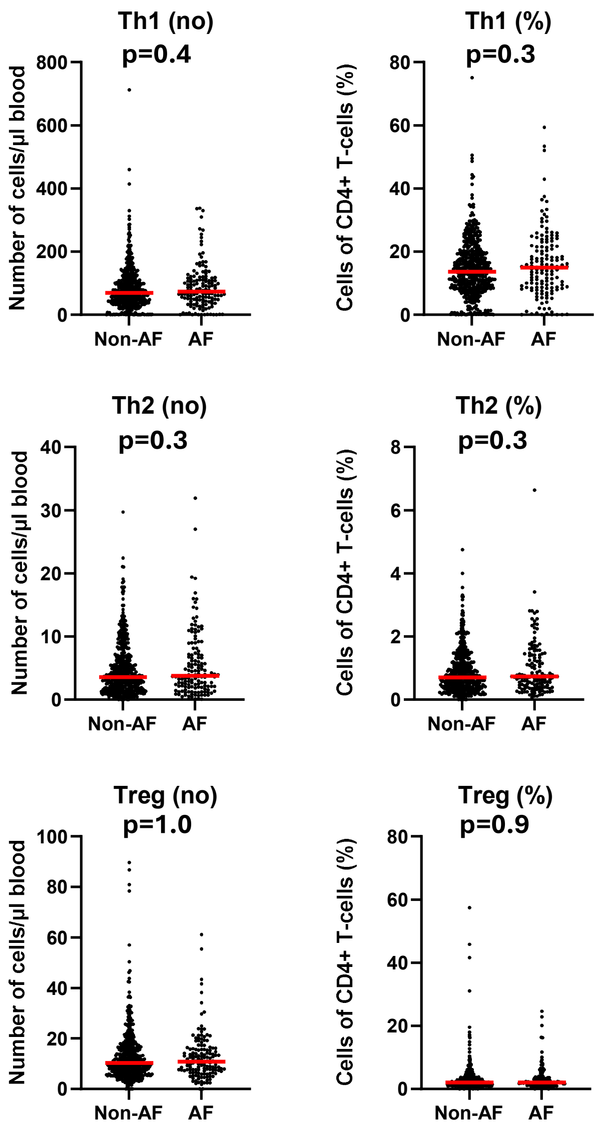

Figure S2. Th1, Th2 and Tregs were analyzed by flow cytometry of cryopreserved mononuclear leucocytes isolated from blood at baseline in individuals who developed atrial fibrillation (AF) during follow-up and in individuals who did not develop atrial fibrillation (non-AF).

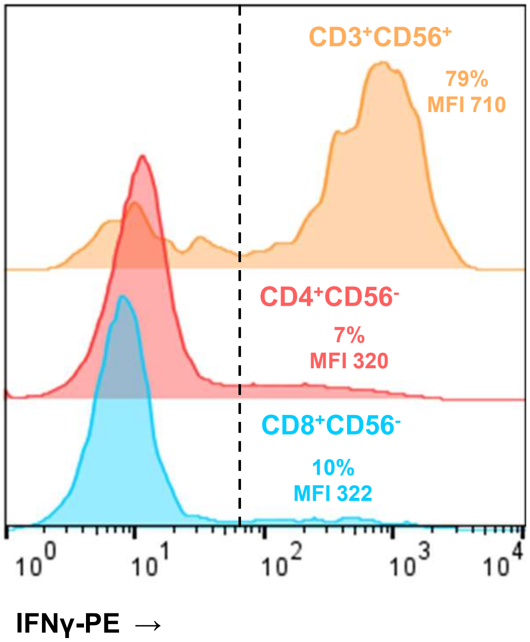

Figure S3. Comparison of IFNγ median fluorescence intensity (MFI) of NKT-like cells, $CD4^+$ T cells and $CD8^+$ T cells.
Mononuclear cells were stimulated with PMA, ionomycin and brefeldin, and IFNγ intensity were measured by flow cytometry in NKT-like cells ($CD3^+CD56^+$), $CD4^+$ T cells ($CD3^+$ $CD4^+CD56^-$) and $CD8^+$ T cells ($CD3^+CD8^+CD56^-$).

Table S1

Baseline levels of T cells in incident AF and non-AF subjects

	Non-AF (n=524)	Incident AF (n=145)	p-value
CD8+ T-cells (no) [1]	279 (188-402)	291 (185-407)	0.5
CD4+ T-cells (no) [1]	551±250	551±266	1.0
CD8+ T-cells (%)[2]	17.6±8.1	18.1±8.6	0.6
CD4+ T-cells (%)[2]	30.4±10.0	29.7±10.4	0.5
CD8+ NKT-like cells (no) [1]	35 (15-81)	49 (21-89)	0.06
CD4+ NKT-like cells (no) [1]	21 (12-36)	23 (13-41)	0.1
CD8+ NKT-like cells (%)[2]	2.2 (0.9-4.3)	2.5 (1.1-5.0)	0.08
CD4+ NKT-like cells (%)[2]	1.2 (0.7-2.1)	1.4 (0.7-2.3)	0.2

[1] Number of cells/µl blood

[2] % of total lymphocytes

Data are presented as median (IQR).

Table S2

Spearman's rho correlation coefficients of NKT-like cells and clinical characteristics

	NKT-like cells (no)	NKT-like cells (%)
Age, y	-0.089*	-0.10**
BMI, kg/m2	0.095*	0.066
HbA1c, %	-0.012	-0.072
Fasting glucose, mmol/L	0.036	0.023
Cholesterol, mmol/L	0.003	-0.015
HDL, mmol/L	-0.11**	-0.083*
LDL, mmol/L	0.005	-0.013
Triglycerides, mmol/L	0.11**	0.065
Systolic BP, mm Hg	-0.051	-0.055
Diastolic BP, mm Hg	-0.018	-0.024
CRP, mg/L	0.033	0.013
eGFR, ml/min pr 1.73 m^2	0.087*	0.070

*p<0.05, **p<0.01

Table S3

Differences in numbers and percentages of $CD3^{+}CD56^{+}$ NKT-like cells according to sex, smoking, diabetes, and history of cardiac events

		NKT-like cells (no)	NKT-like cells (%)
Sex	Men (n=273)	88 (41-174)	5.3 (2.9-10.1)
	Women (n=396)	106 (55-179)*	5.9 (3.2-9.1)
Current smoker	No (n=530)	97 (51-173)	5.9 (3.2-9.7)
	Yes (n=114)	113 (53-192)	5.4 (2.9-8.7)
Diabetes	No (n=542)	95 (51-175)	5.5 (3.1-9.3)
	Yes (n=90)	117 (53-210)	6.5 (3.1-10.5)
History of coronary event	No (n=655)	98 (51-177)	5.8 (3.1-9.3)
	Yes (n=14)	50 (19-148)*	2.9 (1.3-8.2)*

*p<0.05, **p<0.01, ***p<0.001

Variables are presented as median (IQR).

Table S4

Baseline characteristics of incident AF and non-AF in men and women in the Malmö Diet and Cancer cohort

	Men		Women	
	Non-AF (n=206)	Incident AF (n=67)	Non-AF (n=318)	Incident AF (n=78)
Age, years	65.7±1.1	65.7±1.2	65.6±1.1	65.5±1.2
Smoking, %	24.1	6.3**	16.0	17.1
Diabetes mellitus, %	14.5	19.0	12.5	16.9
History of CE, %	4.4	3.0	0.9	3.0
History of stroke, %	0.5	0	0.3	2.5*
History of heart failure, %	1.0	00	0	0
BMI, kg/m^2	26.1±3.5	26.7±3.1	26.0±4.0	27.6±5.0**
HbA1c, %	4.9 (4.6-5.2)	5.0 (4.7-5.4)	5.0 (4.6-5.3)	5.0 (4.7-5.4)
Glucose, mmol/L	5.0 (4.7-5.5)	5.2 (4.7-5.7)	4.9 (4.6-5.4)	5.1 (4.7-5.5)
Cholesterol, mmol/L	5.9 (5.4-6.6)	6.3 (5.6-6.8)	6.7 (6.0-7.4)	6.4 (5.8-7.5)
HDL, mmol/L	1.2±0.3	1.2±0.4	1.5±0.4	1.4±0.3
LDL, mmol/L	4.1±0.9	4.2±1.0	4.6±1.0	4.4±1.1
Triglycerides, mmol/L	1.3 (1.0-1.8)	1.4 (1.0-2.1)	1.2 (0.9-1.7)	1.4 (0.9-1.9)
Systolic blood pressure, mmHg	151±19	153±19	150±20	154±20
Diastolic blood pressure, mmHg	89±10	90±7	88±9	88±9
CRP, mg/L	1.6 (0.7-3.2)	1.3 (0.6-2.8)	1.6 (0.8-3.0)	1.9 (1.0-3.4)
eGFR, ml/min per 1.73 m^2	67 (62-75)	68 (61-75)	65 (59-73)	66 (59-78)

*: p<0.05 in Incident AF vs Non-AF subjects in women.

**p<0.01 in Incident AF vs Non-AF subjects in men or in women.

Table S5

Baseline levels of T-cell subsets in incident AF and non-AF in men and women

	Men		Women	
	Non-AF (n=206)	Incident AF (n=67)	Non-AF (n=318)	Incident AF (n=78)
T-cells	809 (590-1032)	785 (645-974)	918 (710-1183)	1012 (731-1287)
$CD8^+$	280 (171-403)	284 (168-360)	279 (196-398)	321 (185-461)
$CD4^+$	453 (316-647)	450 (273-581)	551 (419-743)	622 (440-771)
Th1 cells	59 (32-94)	56 (31-93)	74 (43-123)	95 (59-122)[#]
Th2 cells	3 (1-5)	3 (1-5)	4 (2-7)	5 (2-8)
Tregs	11 (7-18)	11 (7-16)	10 (6-16)	11 (7-16)
NKT-like cells	84 (40-161)	93 (53-193)	99 (53-177)	121 (74-192)*
$CD8^+$	30 (11-78)	42 (17-97)	38 (18-84)	52 (22-88)
$CD4^+$	19 (9-35)	17 (11-32)	24 (13-38)	32 (17-49)*

Number of cells/µl blood

Data are presented as median (IQR).

*: $p<0.05$ in Incident AF vs Non-AF subjects in women.

[#]: $p=0.075$ in Incident AF vs Non-AF subjects in women.

Table S6
Spearman's rho correlation coefficients of NKT-like cells and clinical characteristics in men and women

	NKT-like cells (no)	
	Men	Women
Age, y	-0.12	-0.071
BMI, kg/m2	0.06	0.11*
HbA1c, %	-0.08	0.016
Fasting glucose, mmol/L	0.08	0.021
Cholesterol, mmol/L	-0.02	-0.016
HDL, mmol/L	-0.16*	-0.14**
LDL, mmol/L	-0.013	-0.008
Triglycerides, mmol/L	0.16**	0.089
Systolic BP, mm Hg	-0.028	-0.068
Diastolic BP, mm Hg	0.03	-0.040
CRP, mg/L	-0.021	0.073
eGFR, ml/min pr 1.73 m^2	0.084	0.095

*p<0.05, **p<0.01

Table S7

Differences in numbers of $CD3^+CD56^+$ NKT-like cells according to smoking, diabetes, and history of cardiac events in men and women

		NKT-like cells (no)	p-value
**Men**			
Current smoker	No (n=210)	87 (41-167)	0.25
	Yes (n=52)	95 (51-208)	
Diabetes	No (n=216)	88 (42-166)	0.27
	Yes (n=40)	114 (41-196)	
History of coronary event	No (n=262)	89 (44-175)	0.027
	Yes (n=11)	30 (17-134)	
**Women**			
Current smoker	No (n=320)	105 (55-176)	0.37
	Yes (n=62)	122 (59-191)	
Diabetes	No (n=326)	102 (55-177)	0.16
	Yes (n=50)	120 (61-227)	

Table S8

Hazard ratios (HR) and 95% confidence intervals (CI) of incident AF by numbers and percentages of NKT-like cells

		HR (95% CI) [2]	p
All	NKT-like cells (no)[1]	1.15 (0.98-1.36)	0.089
Men	NKT-like cells (no)[1]	1.11 (0.85-1.43)	0.45
Women	NKT-like cells (no)[1]	1.26 (1.01-1.58)	0.040
All	NKT-like cells (%)[1]	1.19 (1.01-1.41)	0.044
Men	NKT-like cells (%)[1]	1.14 (0.89-1.44)	0.29
Women	NKT-like cells (%)[1]	1.31 (1.02-1.70)	0.037

[1]Continuous standardized variable.

[2]Adjusted for age, sex, systolic blood pressure, current smoking, diabetes, BMI, triglycerides, HDL, eGFR, prevalent coronary event or stroke, and presence of carotid plaque.
